# Scrotal nodular scabies

**DOI:** 10.1002/ccr3.8542

**Published:** 2024-02-20

**Authors:** Bo Sang, Zehu Liu, Xiujiao Xia

**Affiliations:** ^1^ Department of Dermatology, Hangzhou Third People's Hospital Hangzhou Third Hospital Affiliated to Zhejiang Chinese Medical University Hangzhou China

**Keywords:** mite egg, nodule, scabies

## Abstract

Scabies is a highly infectious parasitic skin disease, The most common lesions are solid and 2–3 mm in diameter (papules). Very few cases will develop into nodular scabies, and microscopy is frequently negative in patients with clinically diagnosed nodular scabies. The accuracy of microscopy depends on the expertise of the operator, particularly in finding burrows and extracting the relevant material.

## Graphical abstract

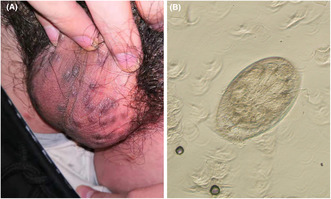

## 1 INTRODUCTION

Scabies is a highly infectious parasitic skin disease which is caused by the mite *Sarcoptes scabiei variety hominis* and spread through person‐to‐person contact. Individuals living in overcrowding, poverty, poor nutritional status, homelessness, dementia, and poor hygiene are susceptible to scabies.^1^ Patients with scabies usually present with intense, refractory, generalized itching that worsens at night. Typical burrows, pruritic papules, and inflammatory nodules are the major manifestations of scabies. Among them, nodular scabies is a severe form of scabies and is relatively rare, only 7% of scabies cases will develop into nodular scabies,^1^ characterized by persistent, hard, erythema, extremely itchy nodules that often involve the genitals.

## 2 CASE REPORT

**FIGURE 1:**
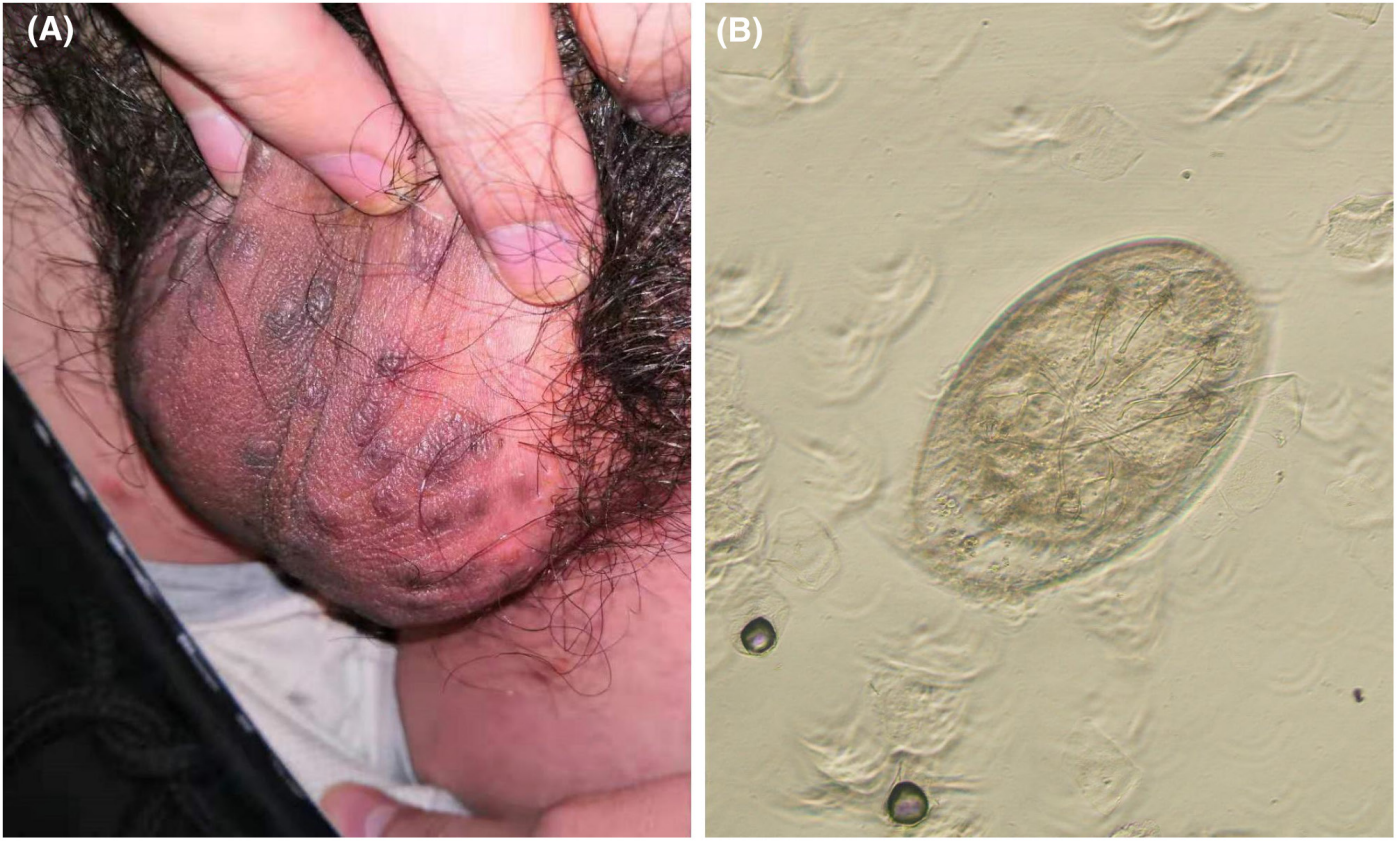
(A) Dense reddish‐brown nodules distributed over the scrotum of a 24‐year‐old male college student. (B) Skin scraping from scrotal nodules upon treatment with 10% potassium hydroxide showing egg (100×).

A 24‐year‐old male college student presented to the dermatology department with a complaint of severe itching of scrotum for last 15 days. The symptoms on the scrotum were preceded by a widespread rash and itching on the trunk, extremities, genital, and hand, which worsened at night. Physical examination revealed reddish papules and vesicles scattered on the trunk and extremities, burrows on the webs between his fingers, and dense reddish‐brown nodules distributed over the scrotum (Figure 1A). Microscopic examination of samples obtained by squeezing the scrotal nodules using a lancet revealed an egg (Figure 1B; 54 × 83 μm in size). The papular lesions resolved in 7 days after treatment with topical compound sulfur cream and oral desloratadine citrate disodium tablets. However, the scrotal nodular lesions persisted in the patient with severe itching. The lesions were then treated with topical steroids and oral anti‐allergy. After an additional 50 days of treatment, the skin lesions entirely disappeared.

## 3 DISCUSSION

The 2020 International Alliance for the Control of Scabies (IACS) Consensus Criteria for the Diagnosis of Scabies include three levels of diagnostic certainty and eight subcategories.^2^ Microscopic observation of mites, eggs, or fecal particles is the way to confirm scabies (level A1). Clinical examination, a history of compatibility and close contact with individuals with similar signs or symptoms or with known scabies can also be used for clinical diagnosis (level B or level C). Though direct microscopic examination of skin scrapings is ideal for identifying the mite and its products, this method has a low positive rate, especially for nodular scabies. Mites and their eggs may rarely be detected from nodular lesions in scabies, and it is thought that nodules usually represent an allergic reaction to mite antigens retained in the body. This case suggests the presence of mites or eggs in nodular scabies, which can be detected under microscopic examination of skin scrapings obtained by mechanical extrusion.

Even after the scabies has been effectively treated, nodular lesions can persist for weeks or months. Hence, the standard approach to treatment is symptomatic treatment of the nodule after scabicidal treatment.^3^ To treat this condition, we need to initiate a regimen of topical and sometimes intra‐lesional corticosteroids and topical calcineurin inhibitors.^3^

## AUTHOR CONTRIBUTIONS

**Bo Sang:** Supervision; validation; visualization; writing – review and editing. **Zehu Liu:** Supervision; validation; visualization; writing – review and editing. **Xiujiao Xia:** Conceptualization; supervision; validation; visualization; writing – original draft; writing – review and editing.

## FUNDING INFORMATION

This work was supported by the Hangzhou Science and Technology Bureau, China (Grant No. 202004A17).

## CONFLICT OF INTEREST STATEMENT

We have no conflict of interest to declare.

## ETHICAL APPROVAL

Ethics approval is not required for de‐identified single case reports based on institutional policies.

## CONSENT

Written informed consent was obtained from the patient to publish this report in accordance with the journal's patient consent policy.

## Data Availability

The data that support the findings of this study are available from the corresponding author upon reasonable request.

## References

[1] Chosidow O . Scabies and pediculosis. Lancet. 2000;355(9206):819‐826. 10711939 10.1016/s0140-6736(99)09458-1

[2] Engelman D , Yoshizumi J , Hay RJ , et al. The 2020 International Alliance for the control of scabies consensus criteria for the diagnosis of scabies. Br J Dermatol. 2020;183(5):808‐820. 32034956 10.1111/bjd.18943 PMC7687112

[3] Mittal A , Garg A , Agarwal N , Gupta L , Khare AK . Treatment of nodular scabies with topical tacrolimus. Indian Dermatol Online J. 2013;4(1):52‐53. 23437425 10.4103/2229-5178.105486 PMC3573456

